# Isolation, Identification, and In Vitro Fungicide Screening of the Pathogen Associated with Pear Dry Blight

**DOI:** 10.3390/pathogens14050432

**Published:** 2025-04-29

**Authors:** Xin Wang, Cai He, Peng Zhang, Lianxin Zhao, Wei Liu, Na Jin, Yanlan Guo

**Affiliations:** 1 Wuwei Academy of Forestry, Wuwei 733000, China; 18909352809@163.com (X.W.); zlx.08.happy@163.com (L.Z.); weiweipeng.3800006@163.com (W.L.); jinna20111@163.com (N.J.); guoyanlan86@163.com (Y.G.); 2 Wuwei Forestry and Grassland Industry Development Service Center, Wuwei 733000, China

**Keywords:** *Diaporthe fukushii*, fungicide sensitivity, pathogen identification, molecular characterisation, pathogenicity, pear dry blight

## Abstract

Pear (*Pyrus* spp.) is a globally important fruit crop, with China leading in the production and cultivation area. Pear dry blight, a destructive fungal disease, has emerged as a significant threat to pear orchards in Wuwei, Gansu Province, China. This study aimed to identify the causal pathogen, evaluate its pathogenicity, and assess the efficacy of commonly used fungicides. A total of 276 fungal isolates were obtained from symptomatic *Pyrus bretschneideri* stems and characterised through morphological and molecular analyses. *Diaporthe fukushii* was identified as the causal pathogen. Pathogenicity assays on Zaosu pear branches and Huangguan pear fruits resulted in 82% and 100% disease incidence, respectively, fulfilling Koch’s postulates. In vitro fungicide evaluations demonstrated that thiophanate-methyl and difenoconazole + propiconazole exhibited the strongest inhibitory effects, followed by mancozeb, metalaxyl-mancozeb, and carbendazim, whereas chloroisobromine cyanuric acid and dimethomorph were the least effective. These findings are critical for developing effective management strategies to mitigate the impact of pear dry blight on pear production.

## 1. Introduction

Pears (*Pyrus* spp.) are the most widely cultivated fruit crops worldwide. China dominates production through its 65.7% contribution to global production and 72.3% of the cultivated area [1]. In 2017, China’s pear orchards covered approximately 1,071,000 hectares, yielding 12.89 million tons [2]. In China, pears hold the third position among fruit crops, following oranges and apples, and the pear industry generates USD 200 million in annual exports [1]. They play a vital role in the rural agricultural economy by supporting livelihoods and providing a stable food source [3]. Moreover, the worldwide demand for pears is increasing due to their rich nutritional profile, which contains high dietary fibre and health-promoting bioactive compounds [4].

Favourable climatic and soil conditions in major pear-producing provinces in China, including Hebei, Shandong, Jiangsu, Anhui, and Gansu, support the cultivation of a wide range of pear varieties, reinforcing China’s status as a centre of origin and genetic diversity for the species [5,6,7]. Recently, Wuwei City, which is located in Gansu Province, has emerged as a key centre for pear cultivation. Pear orchards had expanded to approximately 40,000 hectares in this city due to its temperate continental climate characterised by abundant sunshine (2200–3000 h annually), low rainfall (170 mm per year), and significant temperature variations (average 8 °C) [8].

Although favourable climatic conditions contribute to high fruit quality [9], these same environmental factors also create conditions conducive to pest infestations and disease outbreaks [10], posing significant challenges to pear production and orchard sustainability. Among the most severe threats to Wuwei’s pear industry is pear dry blight, a fungal disease that affects seedlings, stems, and, in severe cases, entire trees. Affected pear plants show symptoms such as leaf wilting and browning, together with branch cankers and shoot dieback, ultimately leading to the death of infected stems or entire trees. Young pear plants are especially affected, which results in significant yield reductions. The disease attacks *P. ussuriensis* Maxim., *P. bretschneideri* Rehd., *P. pyrifolia* Nakai, and multiple other cultivated pear cultivars. Cold regions experience high pear dry blight occurrences, resulting in substantial economic damage by destroying pear orchards [11]. Between 2012 and 2017, the incidence of pear dry blight in Wuwei ranged from 19% to as high as 45% in some orchards, severely impacting local fruit production and industry profitability.

Several fungal pathogens, including *Phomopsis fukushii* (Tanaka & S. Endô), *Diaporthe eres* Nitschke, *D. amygdali* (Delacroix) Udayanga, Crous & K.D. Hyde, *D. longicolla* (Hobbs) J.M. Santos, Vrandecic & A.J.L. Phillips, and *D. neotheicola* A.J.L. Phillips & J.M. Santos, have been associated with pear dry blight. Ding et al. identified *P. fukushii* as the primary pathogen responsible for pear dry blight [12]. However, Guo et al. reported additional pathogenic species associated with infections in different pear cultivars [13]. Pear dry blight has been reported in several Chinese provinces, including Hebei, Henan, Shandong, Shanxi, Jiangsu, Zhejiang, and Yunnan [11], but it had not been documented in Gansu until this study. The expansion of large-scale pear cultivation and the transfer of seedlings from other provinces may have affected the introduction and spread of pear dry blight in Wuwei.

Earlier studies regarding pear dry blight have mainly focused on disease symptoms alongside basic preventative measures, including sanitation practices and chemical treatments [11,14,15,16]. Despite existing research efforts, little is known about how *Diaporthe* species differ pathogenically, how they interact with their environment, and how successful control measures are over extended periods. The Internal Transcribed Spacer (ITS) region of rDNA is the most commonly used genetic marker for fungal species identification due to its high resolution in distinguishing closely related taxa. It has been successfully employed in identifying numerous plant pathogenic fungi, including *Diaporthe* spp. *Colletotrichum* spp., and *Fusarium* spp. [11,13]. The lack of a systematic disease management framework necessitates further research to develop targeted interventions. Given the increasing prevalence of pear dry blight and its impact on pear production, this study aims to isolate and identify the causal pathogen(s) of pear dry blight in Wuwei, assess their pathogenicity, and evaluate their sensitivity to commonly used fungicides. The findings of this study will provide a scientific basis for improved disease management strategies, contributing to the sustainable development of Wuwei’s pear industry.

## 2. Materials and Methods

### 2.1. Sample Collection and Pathogen Isolation

Symptomatic stems of *P. bretschneideri* Rehd were collected from orchards in Wuwei City, Gansu Province, China, over a three-year period from March 2015 to December 2017. The surveyed orchards, aged between 2 and 4 years, were planted with the ‘Huangguan’ cultivar. The surveyed localities and their geographic coordinates are as follows: Xiaoqiba Village in Gaoba Town (102°39′53.47″ E, 37°50′11.35″ N); Henggou Village in Huangyang Town (102°53′13.84″ E, 37°40′24.92″ N); Huazhai Village (102°39′42.58″ E, 37°58′52.82″ N) and Gaolou Village (102°39′16.39″ E, 37°59′56.74″ N) in Zhongba Town; Qijiahu Village in Shuangcheng Town (102°35′41.40″ E, 38°07′30.22″ N); Qianxing Village (102°23′17.46″ E, 37°59′10.12″ N) and Yongfeng Village (102°24′02.15″ E, 37°59′10.50″ N) in Xiying Town; Liugou Village in Xiashuang Town (102°40′28.82″ E, 38°02′21.44″ N); Baiyun Village in Yongchang Town (102°35′31.23″ E, 38°04′41.32″ N); and Shatan Village in Fengle Town (102°23′18.86″ E, 38°04′53.93″ N). Approximately fifty diseased shoots and stem base (five per orchard) were systematically sampled across all orchards to ensure a representative collection of infected plant material. Collected samples were brought to the pathology laboratory of the Wuwei Academy of Forestry Sciences, where some of the samples were visually inspected and observed under a dissecting microscope for the presence of any pathogen propagules or fruiting structures in the infected tissue. Other diseased plant stems showing disease symptoms were washed with sterile water. Infected tissue samples of about 4–5 mm^2^ were excised at the junction of diseased and healthy tissues on the stems using a sterilised blade on a clean, sterilised bench. The excised tissues were surface sterilised in 75% ethanol solution for 30 s, followed by 0.1% mercuric chloride for 5 min, then washed thrice with sterilised water. After sterilisation, samples were air-dried under sterile conditions and placed on Potato Dextrose Agar (PDA; potatoes 200 g, glucose 20 g, agar powder 15–20 g and water to make water to 1000 mL) and Oatmeal Agar (OA; oat 60 g, agar powder 15–20 g, and water to make 1000 mL) in Petri plates and incubated at 25 °C for 3–5 days until fungal colony formation. Emerging fungal colonies were purified through repeated sub-culturing. The colonies were carefully picked and incubated on PDA under the abovementioned conditions. The outer margin of the white hyphae was only transferred on media 3–5 times to ensure purity. Once purified, the fungal strains were grown on slanted PDA and stored at 0 °C for future studies.

### 2.2. Pathogen Morphology

For the morphology characterisation of the pathogen, the purified strains were selected and initially cultured on PDA in Petri plates. After seven days of growth, a 5 mm diameter plug was transferred using a sterilised hole punch and transferred onto fresh PDA and OA in separate Petri plates. Three replicates were performed on each medium. The cultures were incubated at 25 °C under 12 h light and dark cycles. The colony morphology was observed on both OA and PDA every 5 days for a total of 50 days. The isolates were identified based on colony morphology, such as colour, shape, and conidia type. Two hundred conidia from each medium were examined under an Olympus-Cx31rtsf compound light microscope (OLYMPUS, Tokyo, Japan) using clear lactic acid as the mounting medium.

### 2.3. DNA Isolation, PCR and Phylogeny

DNA extraction of morphologically distinguished fungal isolates was performed with the Sangon SK8259 Genomic DNA Extraction Kit (Sangon Biotech Co., Ltd., Shanghai, China). The ITS region was amplified with the universal primers ITS1 and ITS4 (ITS1; 5′-TCCGTAGGTGAACCTGCGG-3′; ITS4: 5′-TCCTCCGCTTATTGATATGC-3′). DNA quality was determined by agarose gel electrophoresis and was quantified using a NanoDrop ND-1000 Spectrophotometer (Thermo Fisher Scientific, Waltham, MA, USA). A final reaction total volume of 25 µL consisting of 2.5 μL 10XPCR buffer, 0.5 μL each of F-primer and R-primer (10 µM each), 0.5 μL genomic DNA, 1 μL of 10 mM dNTP mix, 0.2 μL of Taq polymerase (5 U/µL), and 19.8 μL double distilled water. The PCR procedure started with a 4 min initial denaturation at 94 °C, thereafter 30 cycles consisting of denaturation for 45 s at 94 °C, annealing for 45 s at 55 °C, extension for 1 min at 72 °C, and concluded with a final extension phase at 72 °C for 10 min. In order to view PCR products by agarose gel electrophoresis at a concentration of 1% (*w*/*v*). The concentration of the extracted DNA was determined with a UV-visible spectrophotometer. Sangon Co., Ltd. (Shanghai, China) sequenced the PCR product. The obtained sequences were compared with reference sequences listed in the NCBI GenBank databases via blast searches. The neighbour-joining tree of the aligned sequences was constructed using MEGA 5.0 to compare DNA sequence homology.

### 2.4. Pathogenicity Tests

After molecular identification, the pathogenicity of the purified strains was tested on the current season shoots of Zaosu pear (*P. pyrifolia* Nakai × *P. communis* L.) and the mature fruits of Huangguan pear (*P. bretschneideri* cv. Huangguan). The isolated and purified strain was cultured on PDA at 28 °C for 7 days. A mycelial disc (5 mm in diameter) was taken along the edge of the colony with a sterile puncher for inoculation of fruits and shoots. The stab wound inoculation method was used to determine strain pathogenicity. The purified strain was inoculated separately in healthy branches and fruits. Pathogenicity tests were conducted on the shoots in April 2016 and the fruits in August 2016. For inoculation, each shoot and fruit were wounded using a sterilised knife to peel off a 5 × 5 mm section of epidermal tissue. Fruits and shoot wounds were washed with distilled water and allowed to air dry before inoculation. The mycelial discs were placed onto the wounds, then covered with sterile cotton wool and wrapped with parafilm to prevent drying. Disease symptoms on both fruits and shoots were observed one month after inoculation. A total of 22 shoots or 22 fruits were inoculated with pathogen-containing PDA plugs. For the control treatment, an equivalent number of fruits or shoots with stab wounds were inoculated with sterile PDA plugs. To confirm the causal agent, the pathogenic fungus was re-isolated and identified morphologically based on Koch’s postulates [17].

### 2.5. In Vitro Efficacy of Fungicides Against LGKB-1 Strain

Eight fungicides representative of different chemical groups were tested against the pathogen. Based on the preliminary experiments, different concentrations of each fungicide (Table 1) were incorporated into molten (50 °C) sterile PDA culture media before pouring into Petri plates. Each plate containing fungicide-amended PDA was inoculated with an inverted 10 mm mycelial agar disc from the edge of four-day-old purified fungal cultures. The mycelial discs were placed at the centre of each Petri plate. For the control treatment, plates were prepared by adding sterile distilled water instead of the fungicides to the PDA media. Agar discs free of fungal mycelium were used to inoculate these plates. All plates were subjected to incubation at 28 °C for 7 days. The diameter of colonies on the fungicide-amended media was then compared to the control and recorded, along with percentage inhibition. Each treatment was replicated five times. The diameters of the colonies were measured by cross method, and the relative inhibition rate was calculated using the following formula:

Relative inhibition rate (%) = [(Control colony diameter − Treated colony diameter)/Control colony diameter] × 100%.

### 2.6. Data Analysis

The effective concentration required to inhibit 50% of mycelial growth (EC_50_) was determined for the isolate using a dose–response analysis. Mycelial growth inhibition at different fungicide concentrations was assessed, and the EC_50_ value was estimated using probit regression in IBM SPSS Statistics (version 30.0.0, IBM Corp., Armonk, NY, USA).

## 3. Results

### 3.1. Symptoms

**Figure 1:**
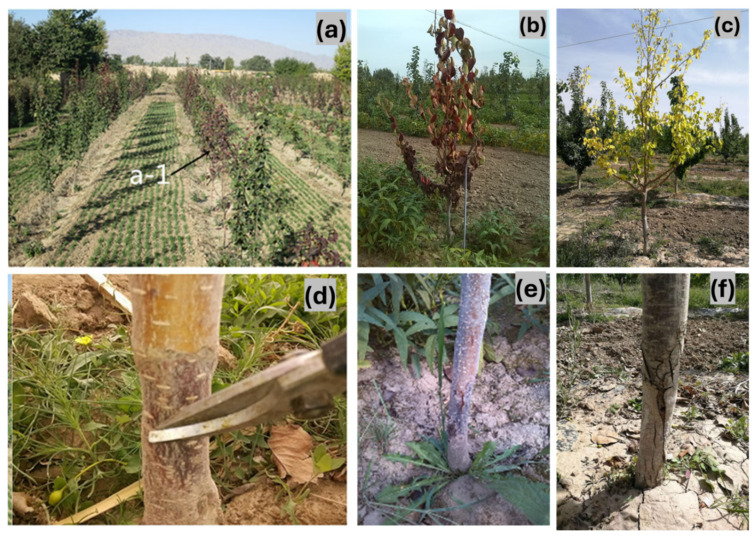
Symptoms of pear dry blight (PDB): (**a**–**c**) symptoms on pear tree leaves (a-1 represented diseased plants in the field. The arrow pointed to the diseased plant with red leaves); (**d**–**f**) symptoms on pear tree stems.

A survey of Wuwei orchards revealed that young orchards between 2 and 4 years old displayed severe disease symptoms across both stems and foliage. While the degree of the infection varied depending on the orchard, in the most affected sites, extensive damage to the stem base and significant leaf wilting was evident, leading to plant decline and, in some cases, mortality (Figure 1). The disease mostly attacked the stem base, where typical symptoms started to show. A dark brown scab formed in the early stages, and the bark progressively blackened. As the infection advanced, the xylem became discoloured, turning brown. The affected bark exhibited signs of decay, including rotting, shrinkage, and sinking, forming an annular contraction pattern. The disease spread upward from the stem base, with cracks appearing at the interface between healthy and diseased tissue. Following disease onset, the current-year leaves of 1- to 2-year-old plants began to wilt, redden, bend, and eventually desiccate and fall. In severe cases, the infection led to complete plant mortality (Figure 1).

### 3.2. Morphological Characterisation

**Figure 2:**
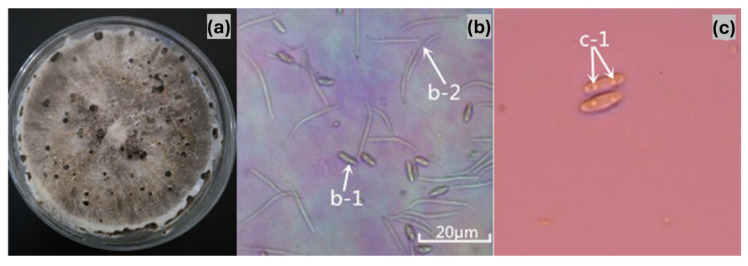
Morphological characteristics of the pear dry blight pathogen. (**a**) Fungal colony on PDA. (**b**) Conidia observed under a microscope (**a**: 40× magnification; b-1: α-conidia; b-2: β-conidia). (**c**) α-conidia structure (c-1: presence of two intracellular oil globules).

Symptomatic tissue samples yielded a collection of 276 fungal isolates. Morphological examination revealed minimal variation among the isolates, and they were identified as belonging to the same fungal species. Colonies with consistent morphology were observed on most isolation plates prepared from infected tissues. All isolates grew well on PDA and OA, with slight variations in growth patterns. The colonies covered 90 mm Petri dishes on PDA within seven days entirely. The substrate mycelium was sparse, moist, and colourless or nearly transparent during the initial 1–4 days, while reproductive mycelium developed after 5–6 days, appearing cottony and white before turning light grey. Pycnidia formed after about 15 days during incubation at 28 °C with a cycle of 12 h of light followed by 12 h of dark. These structures appeared as black, leathery, and hard protuberances in the centre of the colonies. The pycnidia on OA showed similar traits to those on PDA but developed before day 20, while α-type conidia appeared about five days earlier on PDA and were visible around day 20. The presence of β-type conidia at approximately 40 days was a distinguishing characteristic since it appeared exclusively on OA. Only α-type conidia were produced on PDA, whereas both α-type and β-type conidia were present on OA. Based on these morphological characteristics, the pathogen was tentatively identified as *Diaporthe* sp. (Figure 2). Conidial characteristics further supported this identification. The conidiophores were colourless and distinct and showed branching on either one or several axes. With 6–10 × 2–3.5 μm dimensions, the α-type conidia were single-celled, elliptical, and colourless with two separate oil droplets. In contrast, the β-type conidia were single-celled, linear, colourless, and predominantly curved, measuring 12.5–28.5 × 1–2 μm. These morphological traits were consistent with those of the genus *Diaporthe* (Figure 2).

### 3.3. Molecular Characterisation and Phylogenetic Analysis

**Figure 3:**
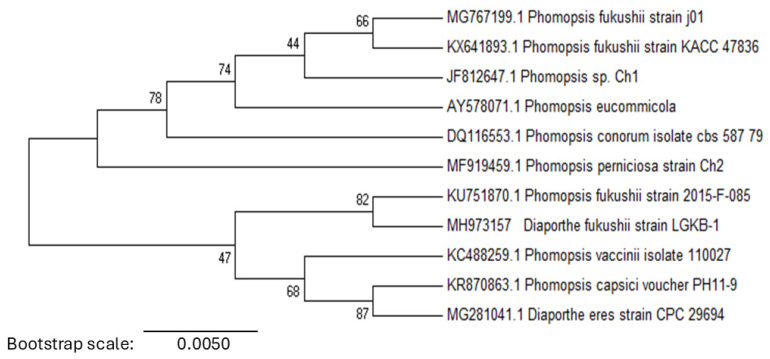
Phylogenetic analysis of pear dry blight pathogen. Neighbour-joining phylogenetic tree based on the ITS region, illustrating the evolutionary relationships among Phomopsis and *Diaporthe* species.

The pathogen’s genomic DNA was amplified using ITS1 and ITS4 primers, yielding a 564 bp sequence. BLASTn analysis revealed a 99% similarity (E = 0.0) to *D. fukushii* (KU751870), indicating a close genetic relationship. Phylogenetic analysis placed LGKB-1 within a well-supported clade alongside *D. fukushii* (KU751870) and *P. fukushii* strains (MG767199, KX641893, KU751870), confirming its genetic affinity (Figure 3). Other related species, including *P. vaccinii* (KC488259), *P. capsici* (KR870863), and *D. eres* (MG281041), formed a distinct lineage within the *Diaporthe*-*Phomopsis* complex. The strong phylogenetic clustering of LGKB-1 with *D. fukushii*, supported by high bootstrap values, further validated its classification. Based on these molecular and morphological characteristics, the pathogen responsible for pear dry blight was identified as *D. fukushii*. The ITS sequence of LGKB-1 was deposited in the GenBank database under accession number MH973157.

### 3.4. Pathogenicity Tests Using LGKB-1 Strain

**Figure 4:**
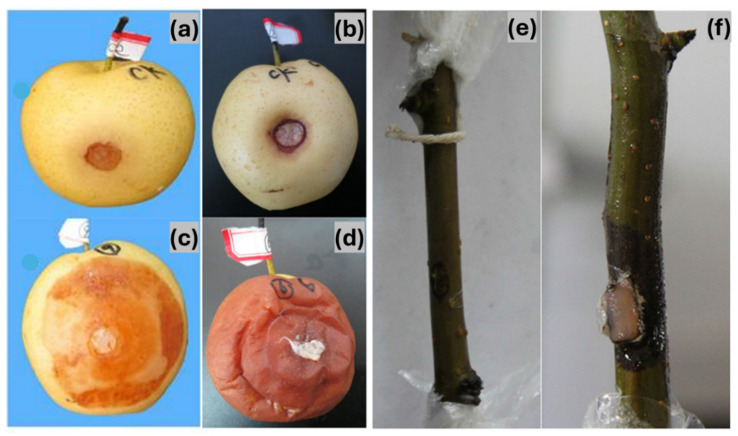
Pathogenicity assessment of pear dry blight (PDB). (**a**,**b**) Control Huangguan pear fruits. (**c**,**d**) Huangguan pear fruits showing disease symptoms after pathogen inoculation. (**e**) Control pear branch. (**f**) Pear branch displaying disease symptoms following pathogen inoculation.

Pathogenicity tests on Zaosu pear branches resulted in a disease incidence rate of 82% (18 out of 22 branches), with symptoms closely resembling those observed in the field (Figure 4f). Mycelial growth was detected on the second day post-inoculation, and by the third day, branches exhibited distinct disease symptoms. As the infection progressed, symptoms extended beyond the inoculation site. In contrast, branches inoculated with sterile plugs remained asymptomatic (Figure 4e). In Huangguan pear fruits, disease symptoms developed three days after inoculation, with a 100% incidence rate (Figure 4c,d). No significant differences in symptom severity were observed among the different isolates. Similarly to the branch inoculations, control fruits remained symptom-free (Figure 4a,b). Pathogen re-isolation from diseased lesions on both branches and fruits yielded isolates identical in morphology to the original inoculum, fulfilling Koch’s postulates and confirming *D. fukushii* as the causal agent of pear dry blight.

### 3.5. Antifungal Effects of Different Fungicides Against LGKB-1 Strain

The in vitro efficiency assays showed that each of the eight fungicides showed different inhibition levels against the pear dry blight pathogen (Table 2). Statistical analysis revealed significant differences in the antifungal activity among the concentrations tested for each fungicide (*p* < 0.005). Based on their EC_50_ values, five fungicides showed strong inhibition. Thiophanate-methyl was the most effective (EC_50_ = 0.25 μg/mL), followed by the difenoconazole + propiconazole combination (EC_50_ = 0.36 μg/mL). Other fungicides with notable inhibitory activity included carbendazim (EC_50_ = 0.98 μg/mL), mancozeb (70%) (EC_50_ = 1.04 μg/mL), and metalaxyl-mancozeb (EC_50_ = 1.51 μg/mL). In contrast, the effects of the remaining three fungicides were comparatively weaker. Metalaxyl-hymexazol had moderate activity (EC_50_ = 16.17 μg/mL), followed by dimethomorph (EC_50_ = 18.55 μg/mL), while chloroisobromine cyanuric acid was the least effective (EC_50_ = 42.36 μg/mL). Regression analysis showed strong linear relationships between fungicide concentration and fungal inhibition, with correlation coefficients ranging from 0.9605 to 0.9982, supporting the reliability of EC_50_ estimates (Table 2).

## 4. Discussion

The present study characterises *D. fukushii* as the causal agent of pear dry blight in Wuwei, Gansu Province, China. Inoculation experiments confirmed its ability to infect pear branches and fruits, demonstrating its pathogenicity and potential for significant yield losses. While *D. fukushii* has previously been reported as a pathogen of *Pyrus* and *Malus* species in China [18,19] and linked to pear shoot canker [20], this is the first study to associate it with pear dry blight in Wuwei. Field observations show that young pear trees become more vulnerable after spring frost injuries, which indicate that environmental stress factors might trigger disease outbreaks [21,22].

The genus *Diaporthe* comprises over 1000 species worldwide, infecting more than 70 plant species, including economically important crops like peach, apple, pear, and mango [23,24,25]. These fungi, with an asexual morph *Phomopsis*, can act as pathogens, endophytes, or saprobes, often causing significant agricultural losses [23]. Due to morphological similarities, molecular characterisation is essential for the accurate identification of species [23,26]. This study used morphological and molecular methods to confirm *D. fukushii* as part of the *D. eres* species complex. Molecular analysis confirmed *D. fukushii* as the pathogen, with 99% sequence similarity to *D. fukushii* and strong phylogenetic clustering within the Diaporthe–Phomopsis complex [23]. This finding aligns with previous research highlighting the genetic diversity and adaptability of *Diaporthe* species in fruit crops [27,28,29,30]. Microscopic analysis identified α-conidia and β-conidia, typical of *Diaporthe* species. While α-conidia were readily observed, β-conidia formed only under specific conditions, requiring prolonged incubation on OA medium at 28 °C with a 12 h light–dark cycle [31].

The rapid onset of symptoms and lesion expansion on pear branches and fruits within three days indicate the efficient colonisation of pear tissues by *D. fukushii*, consistent with the high virulence reported for the *Diaporthe* species [32,33]. The absence of symptoms in control treatments further confirms *D. fukushii* as the primary causal agent.

The evaluation of fungicidal efficacy demonstrated that thiophanate-methyl, mancozeb, metalaxyl-mancozeb, difenoconazole + propiconazole, and carbendazim exhibited strong inhibitory effects against *D. fukushii*. Systemic fungicides such as thiophanate-methyl and difenoconazole + propiconazole disrupt fungal mitosis and ergosterol biosynthesis, making them highly effective [34]. Contact fungicides like mancozeb and metalaxyl-mancozeb also demonstrated strong inhibitory effects, supporting their potential use in integrated disease management programmes. These findings align with previous studies on benzimidazole fungicides, which are known to inhibit β-tubulin polymerisation, and triazole fungicides, which interfere with ergosterol biosynthesis, both essential for fungal development [35].

Chloroisobromine cyanuric acid and dimethomorph exhibited low efficacy, with the former relying on oxidative damage and the latter targeting cellulose biosynthesis. This suggests that *D. fukushii* may have some tolerance to CAA fungicides, but further research is required to confirm this. These findings align with previous studies that report variable [36,37,38,39].

An integrated management strategy incorporating cultural and chemical control measures is essential for effective pear dry blight management. Rotating fungicides with different modes of action is crucial to reducing the risk of resistance [28,40]. The tested fungicides provide a viable chemical control option in orchards where resistant rootstocks are unavailable. Thiophanate-methyl and difenoconazole + propiconazole are the most effective fungicides, while mancozeb serves as a protective treatment. Less effective fungicides, such as chloroisobromine cyanuric acid and dimethomorph, should be used cautiously or in combination with other fungicides to enhance efficacy. Diseased tissues should be excised to the xylem, followed by fungicide application at two-week intervals for three cycles. Covering treated areas with artificial bark has been shown to enhance the persistence and effectiveness of the treatment by protecting it from environmental degradation [41,42]. Additionally, the use of *P. betulifolia* rootstocks has been reported to lower pear dry blight incidence, serving as a preventive measure in newly established orchards [11].

Further research is required to enhance the understanding of *D. fukushii* epidemiology and management in pear orchards. Priority areas include field-based validation of fungicides to assess long-term efficacy, monitoring resistance development, and evaluating environmental factors influencing disease outbreaks, particularly climate-induced susceptibility in young trees. Additionally, genomic studies on virulence factors and host–pathogen interactions may provide insights for developing resistant cultivars.

## 5. Conclusions

This study confirmed *D. fukushii* as the causal agent of pear dry blight in Wuwei, China, through morphological and molecular characterisation. Pathogenicity tests demonstrated that pathogen is highly virulent and causes significant disease incidence in both pear branches and fruits. Fungicide evaluations revealed that thiophanate-methyl and difenoconazole + propiconazole were the most effective treatments, whereas chloroisobromine cyanuric acid and dimethomorph exhibited limited efficacy. An integrated approach incorporating fungicide rotation, cultural practices, and resistant rootstocks to mitigate disease spread and reduce economic losses is recommended.

## Figures and Tables

**Table 1:** Name and details of fungicides used for in vitro testing against fungal pathogens.

Fungicide Name	Concentrations Tested (µg/mL)	Type/Chemical Class	Mode of Action	Manufacturer/Source
Carbendazim (25%)	0.01, 0.1, 1, 10, 100	Systemic/Benzimidazole	Inhibits fungal mitosis	Sichuan Runer Technology Co., Ltd., Chengdu, China.
Chloroisobromine cyanuric acid (50%)	0.01, 0.1, 1, 10, 100	Contact/Halogenated Isocyanurate	Oxidising agent damaging fungal cells	Qingdao Taiyuan Technology Development Co., Ltd. Qingdao, China.
Difenoconazole + Propiconazole (30%)	0.01, 0.05, 0.1, 1, 10	Systemic/Triazole	Disrupts ergosterol biosynthesis	Hebei Guanlong Agrochemical Co., Ltd., Hengshui, China
Mancozeb (70%)	0.01, 0.1, 1, 10, 100	Contact/Dithiocarbamate	Disrupts cell membrane integrity and interferes with enzymes	Sichuan Guoguang Agrochemical Co., Ltd., Chengdu, China.
Metalaxyl-Hymexazol (30%)	0.01, 0.1, 1, 10, 100	Systemic + Contact/Phenylamide + Pyrimidine	Inhibits protein synthesis and disrupts cell division	Changchun Changshuang Pesticide Co., Ltd., Changchun, China
Metalaxyl-mancozeb (58%)	0.01, 0.1, 1, 10, 100	Systemic + Contact/Phenylamide + Dithiocarbamate	Inhibits protein synthesis and disrupts membranes	Shandong Libang Agrochemical Co., Ltd., Heze, China.
Thiophanate-methyl (70%)	0.01, 0.05, 0.1, 1, 10	Systemic/Benzimidazole	Inhibits fungal mitosis	Shandong Zouping Pesticide Co., Ltd., Zouping, China.
Dimethomorph (10%)	0.01, 0.1, 1, 10, 100	Systemic/morpholine derivative	Inhibits fungal cell wall synthesis.	Hebei Zhongbao Green Crop Technology Co., Ltd., Langfang, China.

**Table 2:** Comparative inhibitory effects of eight fungicides on pear dry blight pathogen.

Fungicide	Concentrations Tested (µg/mL)	Regression Equation	Correlation Coefficient	EC50 (μg/mL)
Carbendazim (25%)	0.01, 0.1, 1, 10, 100	y = 5.0112 + 1.0459x	0.9942	0.98
Chloroisobromine cyanuric acid (50%)	0.01, 0.1, 1, 10, 100	y = 4.0216 + 0.6014x	0.9605	42.36
Difenoconazole + Propiconazole (30%)	0.01, 0.05, 0.1, 1, 10	y = 5.5025 + 1.1274x	0.9928	0.36
Mancozeb (70%)	0.01, 0.1, 1, 10, 100	y = 4.9810 + 1.1091x	0.9978	1.04
Metalaxyl-Hymexazol (30%)	0.01, 0.1, 1, 10, 100	y = 4.1422 + 0.7096x	0.9921	16.17
Metalaxyl-Mancozeb (58%)	0.01, 0.1, 1, 10, 100	y = 4.8024 + 1.1031x	0.9927	1.51
Thiophanate-Methyl (70%)	0.01, 0.05, 0.1, 1, 10	y = 5.7143 + 1.2018x	0.9982	0.25
Dimethomorph (10%)	0.01, 0.1, 1, 10, 100	y = 4.1444 + 0.6746x	0.9859	18.55

## Data Availability

All data generated in this study are included in the paper.
